# Knowledge graph embedding for profiling the interaction between transcription factors and their target genes

**DOI:** 10.1371/journal.pcbi.1011207

**Published:** 2023-06-20

**Authors:** Yang-Han Wu, Yu-An Huang, Jian-Qiang Li, Zhu-Hong You, Peng-Wei Hu, Lun Hu, Victor C. M. Leung, Zhi-Hua Du

**Affiliations:** 1 College of Computer Science and Software Engineering, Shenzhen University, Shenzhen, Guang-dong, China; 2 School of Computer Science, Northwesterm Polytechnical University, Xi’an, Shaanxi, China; 3 Xinjiang Technical Institute of Physics and Chemistry, Chinese Academy of Sciences, Ürümqi, China; University of California Irvine, UNITED STATES

## Abstract

Interactions between transcription factor and target gene form the main part of gene regulation network in human, which are still complicating factors in biological research. Specifically, for nearly half of those interactions recorded in established database, their interaction types are yet to be confirmed. Although several computational methods exist to predict gene interactions and their type, there is still no method available to predict them solely based on topology information. To this end, we proposed here a graph-based prediction model called KGE-TGI and trained in a multi-task learning manner on a knowledge graph that we specially constructed for this problem. The KGE-TGI model relies on topology information rather than being driven by gene expression data. In this paper, we formulate the task of predicting interaction types of transcript factor and target genes as a multi-label classification problem for link types on a heterogeneous graph, coupled with solving another link prediction problem that is inherently related. We constructed a ground truth dataset as benchmark and evaluated the proposed method on it. As a result of the 5-fold cross experiments, the proposed method achieved average AUC values of 0.9654 and 0.9339 in the tasks of link prediction and link type classification, respectively. In addition, the results of a series of comparison experiments also prove that the introduction of knowledge information significantly benefits to the prediction and that our methodology achieve state-of-the-art performance in this problem.

## Author summary

The interaction between transcription factors (TFs) and their target genes is a fundamental aspect of transcriptional regulation research, but the number of these interactions that can be studied is currently limited by biological techniques. And the computational methods relevant to the prediction of transcriptional regulation relationships are still not accurate enough and are unable to predict the type of transcriptional regulation interactions. This study presents a multi-task approach namely KGE-TGI for predicting the existence of the interactions between transcription factors and their target genes and the type of them on a knowledge graph. To evaluate our method, we constructed a ground truth dataset and conducted 5-fold cross experiments. The results showed that our method achieved average AUC values of 0.9654 and 0.9339 for link prediction and link type classification, respectively. Comparison experiments also demonstrated that incorporating knowledge information significantly improved performance and our method achieved state-of-the-art results for this problem.

## 1 Introduction

Transcription factors (TFs) are key proteins in mechanisms of gene regulation that function by binding to transcriptional regulatory regions (e.g., promoters, enhancers, and silencers) in genes to control their expression. Usually localized in the 5’-upstream region of target genes, a TF could promote or block the recruitment of RNA polymerase to boost or decrease the transcription rate of genetic information from DNA to mRNA, serving as either an activator or a repressor [1]. An increasing number of TFs have been identified and categorized into different families, of which some are common to several cell types (e.g., AP-1 and NF-*κ* B), whereas others are cell-specific potentially determining the phenotypic characteristics of a cell. A TF can target multiple types of genes while a gene can be also regulated by other functionally similar TFs, forming a complicated and dynamic regulation network. The interactions between TF and their targets lay the important knowledge foundation for deciphering the complex process of gene regulation, and therefore much effort has been made to detect them in research of medical biology and molecular biology.

Existing laboratory techniques developed to identify TF-target gene interactions typically include EMSA [2], ChIP-seq [3], and DAP-seq [4], each with varying utility and distinct strengths and weaknesses. EMSA is used to study the binding pattern of proteins to known DNA oligonucleotide probes, based on the observation that protein-DNA complexes migrate more slowly than free DNA molecules when subjected to non-denaturing polyacrylamide or agarose gel electrophoresis. The chromatin immunoprecipitation (ChIP) method allows analysis of TF–target gene interactions in living cells but requires sequence information of TF and gene as an antibody against the TF and PCR primers for the target DNA sequence must be provided in quantitative PCR. In the process of DAP-seq, TFs are constructed in vitro and bound to target DNA fragments, which are subsequently enriched for analysis. As DAP does not need the specific antibody of the target protein, it has a wider range of applications than ChIP-seq. Despite the great success of laboratory techniques to identify TF-target gene interactions, the results yielded by them are still the tip of the iceberg compared with the complete gene regulation network. In addition, the type of their interaction (activation or inhibition) is largely unknown in the established database. Therefore, there is an urgent need to develop computational approaches to aid the identification of TF-target gene interactions by selecting the most potential pairs for biological assays to verify.

Most of the existing computational methods for identifying interactions between TF and target genes mainly focus on their binding sites, coupled with classical deep learning frameworks like convolutional neural network (CNN) and recurrent neural network (RNN). CNN-based methods use DNA sequence as input data, which is generally treated as image-like matrix and encoded into motif embedding vectors by different types of convolutional kernels [5–8]. In this category of methods, the prediction task of transcription factor binding sites (TFBS) is analogous to image classification, aiming to yield the probability of a binding site at a location of DNA that is scanned. RNN can provide an alternative strategy to achieve the same goal since the DNA sequence is naturally sequential data. Existing methods of this type adopt different RNN variant architectures (e.g., BiGRU and LSTM) to enhance the before-and-after dependency of the features of DNA sequence, solving the long-range dependency problem that CNN meets [9–11]. Though the problem of TFBS prediction has been widely studied with a variety of computational methods proposed, these works can only consider the local structure of DNA sequences and predict the binding probability for each single DNA motif. Their prediction results are of high false-positive rates, partially because they do not consider the general DNA structure and partially because TFBS are often located in the long non-coding sequence. In addition, TFBS prediction cannot infer the interaction type given a pair of TF and gene with their sequences.

To predict TF-target gene interactions directly, some efforts have been made to develop prediction models using different gene expression data sources. The most commonly used biological data is gene expression data, and many classic transcriptional regulatory relationship prediction algorithms have been developed based on this type of data, including GENIE3 [12], NARROMI [13], TIGRESS [14], etc [15, 16]. These methods achieve good results in predicting transcriptional regulatory relationships by performing feature selection and other operations on gene expression data. In addition, there are models such as NetAct [17] that are also based on gene expression data, which can identify the core transcriptional regulatory network and predict network relationships at the same time. On the other hand, Yang et al. leveraged the image data of gene expression generated by the ISH (in situ hybridizations) technique and proposed a residual CNN-based model called GripDL to predict new TF-target gene interactions based on the known ones [18]. With regards to the single-cell RNA-seq (scRNA-seq) data, some tentative ideas have also been put forward recently to solve the same problem at the single-cell resolution. For example, Fan et al. transformed a scRNA-seq expression matrix into a 3D co-expression matrix reflecting gene-gene joint distribution, which was subsequently used for inferring the gene regulation network via 3D CNN [19]. However, neither gene expression image nor scRNA-seq data is expensive and still limited in number, which makes them hard to be widely adopted in real research. With the known TF-target gene interactions increasingly collected from experiments, modeling the known part of GRN and learning its patterns may shed light on the prediction problem for TF-target gene interaction.

Recently, the TRRUST database has been established to include the known human TF-target gene interactions verified by biological experiments [20]. Specifically, it retrieves 9395 human TF-target interactions covering 795 types of human TF, some of which the interaction type is recorded. In mathematics, these interaction data naturally form a graph in which nodes and links indicate TF/target genes and their interactions, respectively, such that existing models based on graph neural networks (GNN) can be applied to. To fill this methodological gap, we previously develop the model of GraphTGI, which simply formulates the GRN as a bipartite graph representing TF and target gene with chemical attribute and DNA sequence as node feature [21]. In this work, we improve it regarding prediction performance and application with a constructed knowledge graph (KG) as base data for information mining. Specifically, multiple types of relational data (including known TF-target gene interactions, GO term annotation, gene-disease association, and chemical-gene interaction), which are intrinsically relevant to GRN, were collected to form the knowledge graph, pushing the quantity limits of GRN mechanisms that are discovered. For each type of subgraph in constructed KG, a single graph neural network was separately established to learn the node representation, which was subsequently integrated to calculate the probability scores for prediction. Considering there are a considerable number of known TF-target gene interactions (46% in the TRRUST database) whose interaction type is yet to be confirmed, the proposed model is designed with a link classification component to predict their type based on the assumption that the unknown link types can be inferred by learning the pattern to the known ones.

This paper is organized into three main sections. In the first section, we provide a detailed description of the KGE-TGI model, including the preprocessing of the data, the link prediction module, the edge type prediction module, and the GradNorm [22] module for adjusting the weights of multiple tasks. In the second section, we present the results and analysis of a series of experiments on the model. These experiments focus particularly on the impact of different graph construction strategies on the performance of the model. Additionally, we conduct a series of ablation experiments to further evaluate the contribution of individual components of the model. The third section discusses the contributions and implications of our work, and provides suggestions for future research. Our model’s contributions can be divided into three parts: (1) To our knowledge, KGE-TGI is the first attempt to construct a transcriptional regulatory knowledge graph by integrating transcriptional regulatory relationship networks and other biological networks to jointly infer potential transcriptional regulatory relationships and predict their types. (2) We used a multi-subgraph convolutional network to learn unique information from different subgraphs and common information from the entire knowledge graph. (3) According to experimental results, our model has demonstrated effectiveness and efficiency in large-scale prediction tasks.

## 2 Methods

### 2.1 Dataset

To perform algorithm evaluation and comparison, we used three databases to construct a multiple relation heterogenous graph as ground truth, which consists of TF nodes, target gene nodes, disease nodes and GO term nodes. We first obtained TF-target gene regulatory relationships from TRRUST database (https://www.grnpedia.org/trrust/), which was manually collected from 11237 PubMed articles [20]. The information of regulation type for each TF-target gene pair is also provided, including activation (33.5%), repression (20.5%) and unknown (46%). The regulatory state of some TF-target gene pairs is dynamic and keeps changing in different biological reactions so that a number of TF-target gene pairs recorded in TRRUST were annotated as both “activation” and “repression”. We secondly obtained the gene-disease associations from DisGeNET database (https://www.disgenet.org/), which integrates data from expert repositories, scientific literature and other publicly available resources [23]. There are 1134942 gene-disease associations covering 21671 genes and 30170 diseases in DisGeNET database. Finally, we retrieved 25826 GO term-TF pairs from GENEONTOLOGY database (http://geneontology.org/) [24].

**Fig 1:**
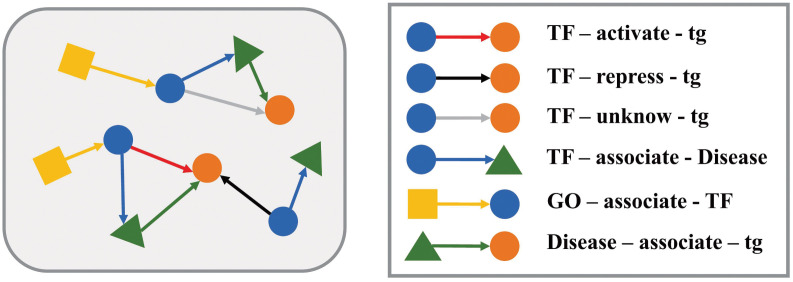
Schematic diagram of the KG of transcriptional regulation.

We consider that the dynamical process of transcriptional regulation not only depends solely on the internal factors such as TFs, but also be influenced by the external factors, especially environmental chemicals. Therefore, we collected chemical-gene associations from Comparative Toxicogenomic Database (CTD, https://ctdbase.org/), which manually curated chemical-gene information from published literatures. CTD database [25] records 124344 chemical-gene associations between 9516 chemicals and 11125 genes, each gene can be associated with 11 different types of chemicals on average. To facilitate the construction of the multiple relation heterogenous graph, we only retained the relationships corresponding to genes whose IDs were matched in all databases of TRRUST, DisGeNET and CTD. As a result, 25,826 experimental verified interactions were used to form the dataset for training and testing our prediction model, relating to 657 TFs, 2146 target genes, 5923 diseases and 4337 GO terms. More details of the dataset are shown in Table 1 and the knowledge graph constructed from the dataset is shown in Fig 1.

**Table 1:** Details of datasets that are used in this work.

	Types	Num.	Resources
Node	TF (TF)	666	TRRUST v.2
	Target gene (tg)	2194	TRRUST v.2
	Disease (D)	5923	CTD
	GO terms (GO)	4337	GENEONTOLOGY
Edge	TF-activate-tg	2897	TRRUST v.2
	TF-repress-tg	1734	TRRUST v.2
	TF-unknow-tg	3907	TRRUST v.2
	TF-associate-D	7775	CTD
	D-associate-tg	31170	CTD
	GO-associate-TF	25826	GENEONTOLOGY

### 2.2 KGE-TGI model

**Fig 2:**
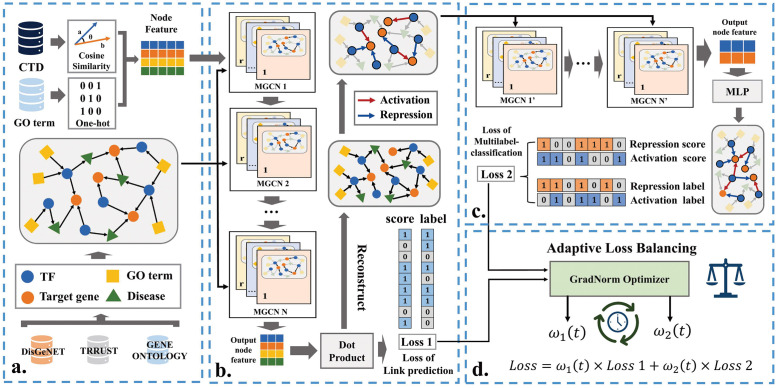
Flowchart of the KGE-TGI model. The model is divided into four parts, including: (a) construction of the knowledge graph of transcriptional regulation from TRRUST, DisGeNET and GENEONTOLOGY databases; (b) a link prediction module, which adopts a MGCNs based model to extract node feature from the knowledge graph and a dot product operation to reconstruct the knowledge graph, and then uses a cross-entropy loss function to calculate the loss of this part; (c) a multilabel classification module, which applies another MGCNs based model to generate node embedding and a MLP layer to predict the transcriptional type of the links, and then uses a multilabel cross-entropy loss function to measure the loss; (d) an adaptive loss balancing module, which uses an independent optimizer to dynamically adjust the balance of two tasks at each training step. And N refers to the number of layers in the proposed model.

The prediction problem is formulated as a multi-task learning problem, in which the goal is to predict the existence of interactions between all types of nodes and the regulation type of TF-target gene interactions simultaneously. As illustrated in Fig 2, KGE-TGI is an end-to-end model that consists of four key components: (a) multiple relation heterogenous graph construction and node feature calculation, (b) Heterogeneous Graph Convolutional Network based module (MGCN) for link prediction (c) MGCN-based module for regulation type prediction and (d) Gradient Normalization (GradNorm) module for adaptive loss balancing in multi-task network.

#### 2.2.1 Multiple relation heterogeneous graph construction

The KGE-TGI model assumes that different biological information networks contain information that can complement each other. For instance, since many diseases arise due to abnormal regulation between genes, we believe that the gene-disease relationship network also holds useful information that can explain the gene-gene relationship network. Additionally, GO terms are a resource used to describe genes and contain valuable information. We constructed a GO term-TF relationship network to introduce this type of information. There are other biological information networks that we did not use, but we believe they also contain valuable information. However, due to resource availability and other reasons, this work only uses disease-related information and GO term-related information for now.

We construct a multiple relation heterogenous graph from the dataset obtained by integrating TRRUST, DisGeNET and GENEONTOLOGY databases. The graph is defined as a directed graph *G* = (*V*, *E*, *T*, *R*), where *V*, *E*, *T*, *R* represent the node set, edge set, node type set and edge type set, respectively. Each node *v* ∈ *V* is associated with a node type *t* ∈ *T*, and each edge *e* ∈ *E* is associated with an edge type *r* ∈ *R*. In our model, the node type set *T* is composed of TFs (*T*_*TF*_), target genes (*T*_*tg*_), diseases (*T*_*D*_) and GO terms (*T*_*GO*_), and the edge type set E is composed of interactions of TF-target gene (*E*_*TF*−*tg*_), target gene-disease (*E*_*D*−*tg*_), TF-disease (*E*_*TF*−*D*_) and GO term-TF (*E*_*GO*−*TF*_). Each node *v*_*i*_(*i* = 1, 2, …, *N*_*n*_) is represented as a feature vector *x*_*i*_, where *N*_*n*_ = |*V*| denote the number of nodes in the graph.

Considering the influence of environmental chemicals on transcriptional regulation, we introduce the information of chemical-gene associations as node features of TF nodes and target gene nodes. Specifically, for each TF type and target gene type node, we use $C=\{c_{1},\ldots ,c_{j},\ldots ,c_{N_{c}}\}$ to denote the relationship between the TF node and all chemicals, where *N*_*c*_ denotes the number of chemicals, and *c*_*j*_ is a binary value indicating whether the specific gene node and the *jth* chemical node are associated. We calculated the cosine similarity between all these chemical relation vectors and store it in a chemical similarity matrix $C_{sim}\in R^{N_{\left(TF+tg\right)}\times N_{c}}$, where *C*_*sim*_(*i*, *j*) denotes the cosine similarity between the *ith* gene node and the *jth* gene node that calculated as follow:

$$\begin{array}{c} C_{sim}\left(i,j\right)=\text{cos}\;\left(C_{i},C_{j}\right)=\frac{C_{i},C_{j}}{\|C_{i}\left\|\times \right\|C_{j}\|} \end{array}$$ (1)

The *C*_*sim*_ matrix is used to as feature of TF and target gene nodes. Besides, we also calculated one-hot encoding of GO term as feature of GO term nodes. And features of other node types are randomly initialized from a normal distribution. It should be noted that our focus in this work is to investigate the impact of biological network topology information on predicting transcriptional regulatory relationships. Unless otherwise stated, KGE-TGI model only employs chemical features as node attributes, and does not utilize gene expression data.

#### 2.2.2 Multi-subgraph convolutional network

The KGE-TGI model assumes that the patterns of information propagation on different types of edges are not totally equivalent, but still have some commonality. Therefore, we use a multi-subgraph convolution network (MGCN) to simultaneously extract node features unique to different types of edges and node features common to all types of edges.

**Fig 3:**
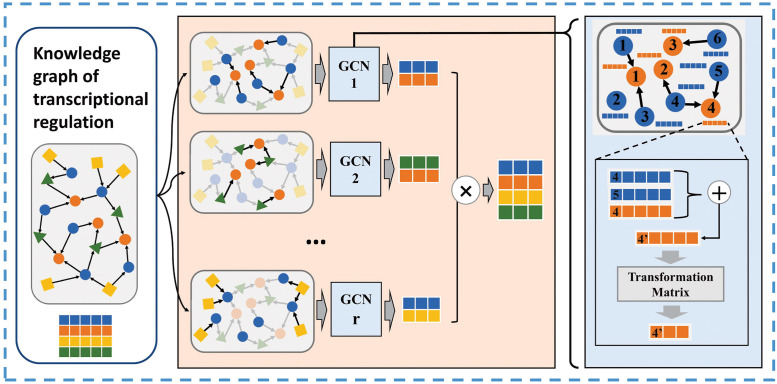
Schematic diagram of the multi-subgraph convolution network. Taking the knowledge graph of transcriptional regulation as input, the multi-subgraph convolution network divides the graph into multiple subgraphs according to different relationships, and uses multiple independent GCNs to extract features separately, and then concatenates all the features together.

As shown in Fig 3, the module treats the graph as |*R*| subgraphs based on the edge type, each subgraph only contains edges of one type. Then a dependent graph convolution kernel is used to extract node features on each subgraph, which reads the features from source nodes and writes the updated ones to destination nodes. If these subgraphs have the same destination node, the results of convolution are aggregated by summing up. The process of node feature update on the r type subgraph is as follows:

$$\begin{array}{c} h_{i}^{r^{\prime }}=\sum_{j\in N_{i}^{r}}W_{r}h_{j}^{r}+b^{r} \end{array}$$ (2)

where $h_{i}^{r^{\prime }}$ is the output feature of the *ith* node generated on the subgraph of *r* edge type, $N_{i}^{r}$ is the set of nodes adjacent to the *ith* node on the *r* type subgraph, $h_{j}^{r}$ is the origin feature of the *jth* node, $W_{r}\in R^{d_{j}\times d_{i}^{\prime }}$ is the transformation weight matrix of the *r* edge type subgraph, and *b*^*r*^ is the bias. *d*_*j*_ is the dimension of input feature of *jth* node and $d_{i}^{\prime }$ is the dimension of the embedding set by the model. We sum up the results to aggregate the features extracted from different subgraphs as follows:

$$\begin{array}{c} h_{i}^{\prime }=\sigma \left(\sum_{r\in R}h_{i}^{r^{\prime }}\right) \end{array}$$ (3)

where $h_{i}^{\prime }$ is the final feature of the *ith* node and *σ* is the activation function to provide nonlinearity. The MGCN module is a general framework that can aggregate features with different dimensions automatically.

#### 2.2.3 Link prediction module

We first describe the module used in the link prediction task. As shown in Fig 2(b), the link prediction module takes the whole heterogeneous graph and all origin node features as input, uses MGCN module to extract features and generates the embedding of each node. Taking the embedding of all nodes as input, the module performs a dot product operation to calculate the probability between each pair of nodes as follows:

$$\begin{array}{c} P_{i,j}=sigmoid\left(h_{i}^{T}\cdot h_{j}\right)=\frac{1}{1+exp(-h_{i}^{T}\cdot h_{j})} \end{array}$$ (4)

where *P*_*i*,*j*_ is the probability between the *v*_*i*_ and *v*_*j*_ node, and sigmoid function is used to map the value to the range of [0, 1]. If *P*_*i*,*j*_ > 0.5, the *ith* and *jth* node are considered to be linked, otherwise they are considered to be unlinked. Using the probability *P*_*i*,*j*_ and the edge label, KGE-TGI model applies a cross-entropy function to calculate the loss of the link prediction module as follows:

$$\begin{array}{c} L_{1}=\frac{-1}{N_{n}}\sum_{i,j\in V}({\hat{P}}_{i,j}log\left(P_{i,j}\right)+\left(1-{\hat{P}}_{i,j}\right)log\left(1-P_{i,j}\right) \end{array}$$ (5)

where *V* is the set of all nodes, and ${\hat{P}}_{i,j}$ is the label of the edge between *ith* node and *jth* node.

#### 2.2.4 Regulation type prediction module

The link prediction module reconstructs the heterogeneous graph by calculating the probability of existence of edges between all nodes. By additionally introducing regulatory type information, the TF-target gene edges in the graph are divided into 2 categories: activation and repression. Instead of taking the whole reconstructed graph directly, the regulation type prediction module only takes the subgraph consisting of edges of activation and repression as input. The module applies another MGCN to extract features from the subgraph, and then uses a multi-layer perceptron to predict the regulation type of each edge as follows:

$$\begin{array}{c} y_{ij}^{1},y_{ij}^{2}=softmax\left(W_{2}\times \left(h_{i}\|h_{j}\right)\right) \end{array}$$ (6)

where $y_{ij}^{1}$ and $y_{ij}^{2}$ are the scores of the *ith* and *jth* node being activated and repressed respectively, $W_{2}\in R^{2d_{embedding}\times 2}$ is a trainable weight matrix, || is the concatenation operation, and *d*_*embedding*_ is the embedding feature size of nodes. The multi-label cross entropy loss function is utilized to evaluate the differences between the predicted type $\hat{y}$ and the ground truth type *y* of size (*N*_*TF*+*tg*_, 2) as follows:

$$\begin{array}{c} L_{2}=\frac{-1}{2}\sum_{k}y\left[k\right]log\left({\left(1+exp\left(-\hat{y}\left[k\right]\right)\right)}^{-1}\right)+\left(1-y\left[k\right]\right)log\left(\frac{exp(-\hat{y}\left[k\right])}{1+exp(-\hat{y}\left[k\right])}\right) \end{array}$$ (7)

where $k\in \{0,\ldots ,N_{\left(TF+tg\right)}-1\}$ is the subscript of *y* and $\hat{y}$, and *y*[*k*] ∈ {0, 1}.

#### 2.2.5 Gradient normalization module

We use a multi-task model to predict whether the transcriptional regulatory relationship exists and the type of the regulatory relationship, using the complementary information of the two tasks to improve the performance, generalization ability and robustness of the model. However, in the training process, the different tasks of the multi-task network need to be appropriately balanced, so as to ensure that the overall parameters of the network can converge in the direction that all tasks can achieve better performance. Different tasks of the loss function will produce different gradients, which will cause the update of the network parameters of different tasks to be unbalanced in the back propagation process. If the gradient produced by the loss function of one task dominates, then the network parameters of this task will be more likely to converge to a better state, while the network parameters of the other task will be more likely to be ignored.

To solve this problem, we introduce a multi-task loss balance algorithm GradNorm [22], an effective method to adaptively adjust the balance between the two tasks. For the loss function of the KGE-TGI model $L\left(t\right)=\sum \omega_{i}\left(t\right)L_{i}\left(t\right)$, GradNorm aims to learn the function $\omega_{i}\left(t\right)$ to dynamically adjust gradient norms, so that all tasks could be trained at similar rates. We first describe the relevant parameters as follows:

$$\begin{array}{c} G_{W}^{\left(i\right)}={\left\|\Delta_{W}\omega_{i}\left(t\right)L_{i}\left(t\right)\right\|}_{2} \end{array}$$ (8)

where W denotes the weight layers shared by all modules, and the formula denotes the L2 norm of the gradient using single-task loss $\omega_{i}\left(t\right)L_{i}\left(t\right)$ for W layer at training time t.

$$\begin{array}{c} {\bar{G}}_{W}\left(t\right)=Avg\left(G_{W}^{\left(i\right)}\left(t\right)\right) \end{array}$$ (9)

where ${\bar{G}}_{W}\left(t\right)$ is the average gradient norm for all tasks. For each task, GradNorm calculate various training rate as follows:

$$\begin{array}{c} L_{i}\left(t\right)=\frac{{\tilde{L}}_{i}\left(t\right)}{L_{i}\left(0\right)} \end{array}$$ (10)

$$\begin{array}{c} r_{i}\left(t\right)=\frac{{\tilde{L}}_{i}\left(t\right)}{Avg({\tilde{L}}_{i}\left(t\right))} \end{array}$$ (11)

where ${\tilde{L}}_{i}\left(t\right)$ is the loss ratio for task *i* at time t, $Avg({\tilde{L}}_{i}\left(t\right))$ is the average loss ratio across all tasks, and $r_{i}\left(t\right)$ is the relative inverse training rate for task *i*, which is used to balance the gradients. Specifically, the higher the value of the $r_{i}\left(t\right)$, the higher the weight of task *i* loss should be. Finally, GradNorm calculates *L*_*grad*_ as a loss dedicated to updating $\omega_{i}\left(t\right)$, which is defined as follows:

$$\begin{array}{c} L_{grad}=\sum_{i}{\left|G_{W}^{\left(t\right)}-{\bar{G}}_{W}\left(t\right)\times {\left[r_{i}\left(t\right)\right]}^{\alpha }\right|}_{1} \end{array}$$ (12)

where *α* is an hyperparameter to set the strength of adjustment. Concretely, we perform GradNorm in our model follow these steps: (1) initialize all *ω*_*i*_(0) to 1 and initialize weights of network, (2) set *α* to 1.5 and pick the weight layer W which are shared between tasks, (3) take input data to perform a standard forward pass and calculate the total loss $L\left(t\right)=\sum \omega_{i}\left(t\right)L_{i}\left(t\right)$ at each train step, (4) compute $G_{W}^{\left(i\right)}\left(t\right)$, $r_{i}\left(t\right)\;\forall \;i$ and ${\bar{G}}_{W}\left(t\right)$, (5) compute *L*_*grad*_ and use it to compute GradNorm gradient $\Delta_{\omega_{i}}L_{grad}$, meanwhile keeping ${\bar{G}}_{W}\left(t\right)\times {\left[\nabla_{i}\left(t\right)\right]}^{\alpha }$ constant, (6) update $\omega_{i}\left(t\right)$ to $\omega_{i}\left(t+1\right)$ by using $\Delta_{\omega_{i}}L_{grad}$, (7) update weights of whole model using $\Delta_{weights}L\left(t\right)$, which is a standard backward pass, and (8) renormalize $\omega_{i}\left(t+1\right)$ to make sure $\sum_{i}\omega_{i}\left(t+1\right)=N_{task}$ where *N*_*task*_ is the number of tasks.

## 3 Results

### 3.1 Evaluation criteria

The proposed KGE-TGI model is evaluated on a multi-subgraph constructed from three databases, namely, TRRUST, DisGeNET and GENEONTOLOGY. To evaluate the quantitative performance of the KGE-TGI model, we have used two sets of evaluation criteria for the task of regulation interaction prediction and regulation type prediction respectively. The first set of evaluation criteria includes accuracy, precision, recall, F1-score and AUC. And the second one includes average per-class precision (CP), recall (CR), F1-score (CF1), Hamming loss and AUC. We defined the evaluation criteria in the following.

$$\begin{array}{c} Acc=\frac{TP+TN}{TP+TN+FP+FN} \end{array}$$ (13)

$$\begin{array}{c} Pre=\frac{TP}{TP+FP} \end{array}$$ (14)

$$\begin{array}{c} Recall=\frac{TP}{TP+FN} \end{array}$$ (15)

$$\begin{array}{c} F1-score=\frac{2\times P\times R}{P+R} \end{array}$$ (16)

$$\begin{array}{c} HL=\frac{1}{N_{classes}}\sum_{j=0}^{N_{classes}-1}1\;\left({\hat{y}}_{j}\neq y_{j}\right) \end{array}$$ (17)

$$\begin{array}{c} CP=\frac{1}{|E_{all}|}\sum_{e\in E_{all}}\frac{|y_{e}\left|\cap \right|{\hat{y}}_{e}|}{{\hat{y}}_{e}} \end{array}$$ (18)

$$\begin{array}{c} CR=\frac{1}{|E_{all}|}\sum_{e\in E_{all}}\frac{|y_{e}\left|\cap \right|{\hat{y}}_{e}|}{y_{e}} \end{array}$$ (19)

$$\begin{array}{c} CF1=\frac{1}{|E_{all}|}\sum_{e\in E_{all}}\frac{2\times \frac{y_{e}\cap {\hat{y}}_{e}}{|{\hat{y}}_{e}|}\times \frac{y_{e}\cap {\hat{y}}_{e}}{|y_{e}|}}{\frac{y_{e}\cap {\hat{y}}_{e}}{|{\hat{y}}_{e}|}+\frac{y_{e}\cap {\hat{y}}_{e}}{|y_{e}|}} \end{array}$$ (20)

where *TP*/*TN* and *FP*/*FN* denotes the number of positive/negative results that correctly indicated and wrongly indicated, respectively. *P*/*R* represents the precision score and the recall score, *E*_*all*_ represents the set of samples, *y*_*e*_ and ${\hat{y}}_{e}$ represents the subset of *y* and $\hat{y}$ with sample *e*. *HL* denotes Hamming loss, which is the fraction of the wrong labels to the total number of labels.

In each fold, we also computed the Receiver Operating Characteristic (ROC) curve and the Area Under the Curve (AUC) for each task module. We first computed the corresponding true positive rates (TPRs) and false positive rates (FPRs) for each threshold value, and then plotted the ROC curve by plotting the TPRs versus FPRs. The AUC value was used to measure the comprehensive performance of the KGE-TGI model. AUC = 0.5 indicates that the KGE-TGI model is no better than random guessing and AUC = 1 indicates perfect prediction. In this paper, We use AUC1 and AUC2 to represent the AUC values of link prediction task and link type classification task, respectively.

In fact, it is difficult to fully verify that there is no relationship between a pair of genes in biological experiments, so there is a lack of negative sample data to describe the gene regulatory network. To be able to validate the model performance, we constructed a negative sample set of the gene regulatory network by randomly sampling from unlabeled data, the size of which is the same as the positive sample dataset. We assume that the probability of sampling a positive sample in the unlabeled data is very small, and we also re-sample the negative sample data in each forward pass to further avoid sampling positive samples.

We trained our model for 300 epochs with a learning rate of 0.001 and a weight decay of 0.0001. We chose LeakyReLU function to save computational resources and avoid the gradient vanishing problem, and the slope value for LeakyReLU was set to 0.2. The KGE-TGI model also adopts a dropout strategy to avoid overfitting problem and sets the drop-out rate to 0.2. The parameters of the KGE-TGI model were initialized by the Xavier initialization method and optimized by the Adam optimizer.

We tested the performance of the proposed model using k-fold (k = 2, 5, 10) cross-validation. Specifically, all the samples are divided into k equal-sized groups, in which each group is used as the test set in turn and the others are used as the training set. The average performance of the model is reported in Table 2.

**Table 2:** Prediction performance of KGE-TGI in K-fold cross-validation.

*K*	AUC1	Acc	Pre	Recall	F1
2	0.9251	0.8537	0.8727	0.8282	0.8499
5	0.9654	0.9231	0.8956	0.9579	0.9257
10	**0.9724**	**0.9350**	**0.9070**	**0.9694**	**0.9371**
*K*	AUC2	HL	CP	CR	CF1
2	0.9009	0.1439	0.8825	0.8363	0.8517
5	0.9339	0.0897	0.9413	0.8996	0.9135
10	**0.9348**	**0.0809**	**0.9539**	**0.9114**	**0.9526**

### 3.2 Effect of using different graph construction strategies

**Fig 4:**
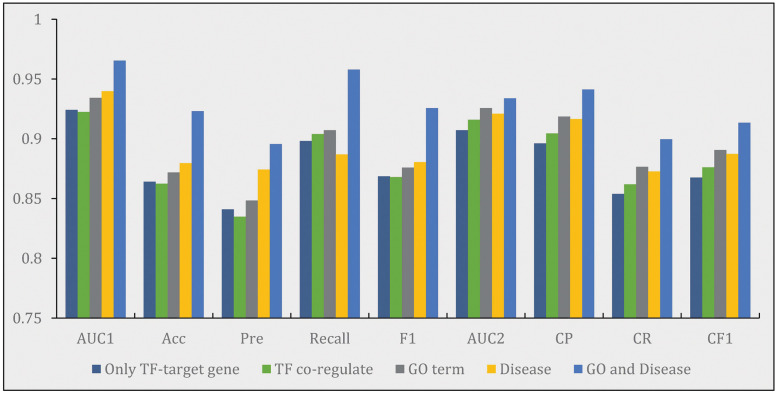
**Performance comparison of KGE-TGI model using different graph construction strategies**, including: (i) only using the data of TF-target gene pairs as a baseline for graph construction; (ii) adding TF co-regulate data to the baseline; (iii) adding additional GO terms-related data to the baseline; (iv) adding additional disease-related data to the baseline; (v) adding both disease-related data and GO terms-related data to the baseline.

We believe that the complexity of the transcription regulatory network is not only determined by the relationship between genes, but also affected by external factors. Therefore, we introduce chemical information, disease information, GO term information as supplements to construct a multi-relational attributed gene regulatory graph. We believe that the abnormal gene transcription regulation process can lead to complex diseases. Therefore, the gene-disease relationship network contains information that can supplement the prediction of transcription regulation relationships. In addition, GO terms, as a resource for describing genes, carry a lot of information about genes themselves. Therefore, we constructed a transcription regulation knowledge graph containing gene-disease relationships and GO term-TF relationships to improve the prediction performance of the model. To explore the impact of different relationships on the model, we compared the performance of KGE-TGI model with five different graph construction strategies, including the following: (i) only using TF-target gene pairs data as a baseline for graph construction; (ii) adding TF co-regulate data to the baseline; (iii) adding GO term-TF data to the baseline; (iv) adding disease-related data to the baseline; (v) adding both GO term—TF data and disease-related data to the baseline. The experiments were tested under 2 MGCN layers upon 5-fold cross-validation. The results were reported in Fig 4.

From Fig 4, we can see that the performance of KGE-TGI model using the base graph is the worst, and the performance of the model using the base graph with TF co-regulate edges is similar to it. Strategies (iii) and (iv) have proved to be helpful for improving the performance on both link prediction task and link multilabel-classification task, which indicates that the information of GO terms and related diseases are the useful complementary information for revealing the mechanism of relationships among genes. The results also show that the performance of the KGE-TGI model using strategy (v) is outstanding, which attests to the assumption that the external factors do have a significant impact on the regulatory network. Base on the results, we anticipate that the performance of the KGE-TGI model will be further improved when more comprehensive external information is introduced in the future.

### 3.3 Comparison of model parameters

The parameters of the KGE-TGI model include depth of model, width of model and the convolution kernel used in the model. The depth of the model refers to the number of layers, and the width of the model refers to the dimension of the embedding vector.

#### 3.3.1 Depth of model layer numbers

Setting embedding dimension of link prediction module and link classification module to 256 and 32, we tested the KGE-TGI model with 1, 2 and 3 MGCN layers for 5-fold cross-validation and the results are shown in Table 3. From Table 3, we can see that the performance of the KGE-TGI model with 2 MGCN layers is the best with regard to both tasks on all metrics. With the increase of the number of layers, the performance of the model is shown to degrade, which may be due to the gradient vanishing problem.

**Table 3:** Prediction performance of KGE-TGI model with different numbers of MGCN layers.

Layer	AUC1	Acc	Pre	Recall	F1
1	0.9274	0.8504	0.8419	0.8630	0.8521
2	**0.9654**	**0.9231**	**0.8956**	**0.9579**	**0.9257**
3	0.9273	0.8403	0.8932	0.7732	0.8287
Layer	AUC2	HL	CP	CR	CF1
1	0.9158	0.1261	0.8986	0.8548	0.8694
2	**0.9339**	**0.0897**	**0.9413**	**0.8996**	**0.9135**
3	0.9260	0.0982	0.9337	0.8873	0.9028

#### 3.3.2 Embedding size of KGE-TGI model

To explore the impact of the embedding dimension on the performance of the KGE-TGI model, we set the embedding dimension of the link prediction module to 32, 64, 128, 256 and 512 for testing. The link classification module takes the output of the link prediction module as input, so that the embedding dimension of the former corresponds to the embedding dimension of the latter, and was set to 4, 8, 16, 32 and 64 in the experiments. As shown in Table 4, the performance of the KGE-TGI model improves as the embedding dimension increases from 32 to 256, and then decreases slightly as the embedding dimension continues to increase.

**Table 4:** Prediction performance of KGE-TGI with different embedding size.

Layer	AUC1	Acc	Pre	Recall	F1
64	0.9427	0.8852	0.8432	0.9466	0.8919
128	0.9542	0.9036	0.8697	0.9496	0.9078
256	**0.9654**	**0.9231**	0.8956	**0.9579**	**0.9257**
512	0.9635	0.9163	**0.9107**	0.9233	0.9168
Layer	AUC2	HL	CP	CR	CF1
8	0.9181	0.1110	0.9104	0.8689	0.8827
16	0.9159	0.1092	0.9148	0.8728	0.8868
32	**0.9339**	0.0897	0.9413	0.8996	0.9135
64	0.9335	**0.0895**	**0.9414**	**0.9001**	**0.9139**

#### 3.3.3 Performance comparison of different neural network-based methodologies

We also tested the performance of the link prediction module with different graph neural networks, including Graph Convolutional Networks (GCN), GraphSAGE [26], Graph Attention Networks (GAT) [27], EdgeConv [28] and Graph Isomorphism Network (GIN) [29]. As shown in Table 5, using GCN kernel achieves the best performance on AUC, accuracy, recall and F1 scores, while using GraphSAGE achieves the best performance on precision. KGETGI model improves 9% in AUC performance compared to GraphTGI model, indicating the effectiveness of multi-relation knowledge graph and multi-subgraph convolution network architecture. For the link multilabel classification module, we compared different type of neural networks, including GCN, Convolutional Neural Network (CNN) and Multilayer perceptron (MLP). As shown in Table 6, the performance of the link classification module using GCN is the best, which indicates that the graph structure information of regulatory network is also useful to the link type classification task.

**Table 5:** Prediction performance of link prediction module with different GNN layer.

GNN	AUC1	Acc	Pre	Recall	F1
GAT	0.8289	0.7991	0.8216	0.7647	0.7921
EdgeConv	0.6244	0.5761	0.5593	0.5524	0.4937
GINConv	0.5446	0.5447	0.5430	0.6644	0.5608
GraphTGI	0.8864	0.7989	0.7996	0.7986	0.7986
GraphSAGE	0.9285	0.7764	**0.9148**	0.6097	0.7312
KGE-TGI	**0.9654**	**0.9231**	0.8956	**0.9579**	**0.9257**

**Table 6:** Prediction performance of multilabel classification module with different neural layer.

Network	AUC2	HL	CP	CR	CF1
CNN	0.6270	0.3801	0.5141	0.4803	0.4916
MLP	0.6638	0.3262	0.6738	0.6323	0.6461
KGE-TGI	**0.9339**	**0.0897**	**0.9413**	**0.8996**	**0.9135**

### 3.4 Prediction performance of KGE-TGI model using different datasets

To validate the effectiveness of the model on other datasets, we compared its performance on hTFtarget [30], TFLink [31], and regNetwork [32]. As shown in the Table 7, the performance varied across different datasets, with the worst performance on hTFtarget, possibly due to the limited number of recorded transcription factors and relationships in the hTFtarget dataset.

**Table 7:** Prediction performance of KGE-TGI model using different datasets in 5-fold cross validation.

dataset	AUC	Acc	Pre	Recall	F1
hTFtarget	0.7626	0.6932	0.6655	0.7769	0.7169
TFLink	0.8723	0.7856	0.7940	0.7713	0.7825
regNetwork	0.9069	0.8223	0.8303	0.8103	0.8200
TRRUST	0.9654	0.9231	0.8956	0.9579	0.9257

### 3.5 Performance comparison of KGE-TGI model with other methods

**Fig 5:**
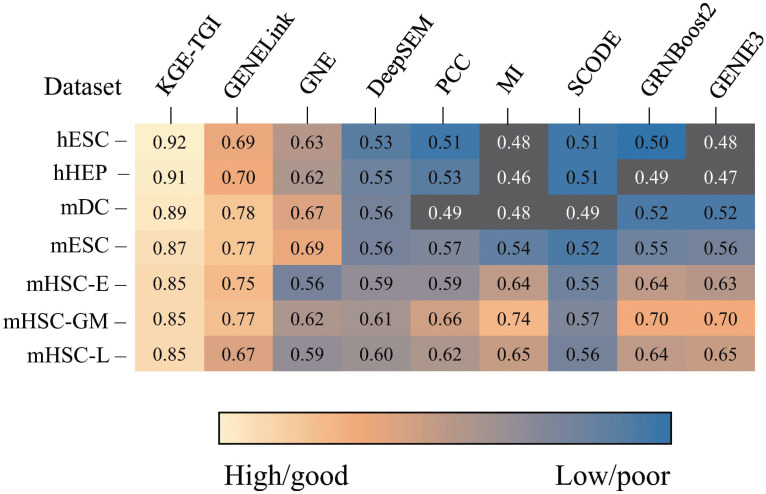
Summary of the GRN prediction performance in terms of AUC value. The dark squares denote performance worse than random predictors.

To better explore the performance of our model, We compared the AUC values of the KGE-TGI model with those of other models. We use the Non-specific ChIP-seq data corresponding to TRRUST as the input of these models, and experiment with 1000+ TFs. Specifically, in order to compare KGE-TGI model with other models more fairly, we used non-specific ChIP-seq gene expression data as the input node features for KGE-TGI model, instead of using chemical features as node features. We directly compared the result of KGE-TGI model with the results of other methods recorded in the paper by Guangyi Chen et al. [33], as shown in the Fig 5.

As shown in the figure, the results demonstrate that the KGE-TGI model outperforms other models on all datasets. We believe that the reason for the better performance of our model is that the transcriptional regulatory knowledge graph we constructed contains richer information, which also indicates that our model’s basic assumptions are correct. By using multi-subgraph graph convolutional operations, more useful complementary features can be learned from different biological network information, thereby improving the predictive ability of the model.

### 3.6 Performance comparison of KGE-TGI model with or without GradNorm algorithm

**Fig 6:**
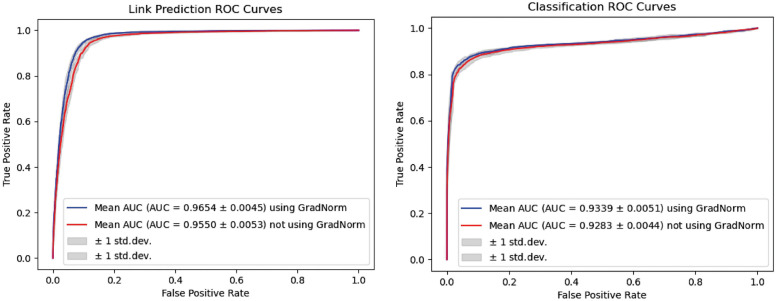
The ROC curves yielded by KGE-TGI model with or without GradNorm algorithm under 5-fold cross-validation.

To verify the performance improvement of GradNorm, we compared the performance of the model with GradNorm and the model with fixed loss weights, which is set to 1. Fig 6 shows the ROC curves of the two modules, and the results show that the model with GradNorm achieves better performance than the model with fixed loss weights on both tasks.

**Fig 7:**
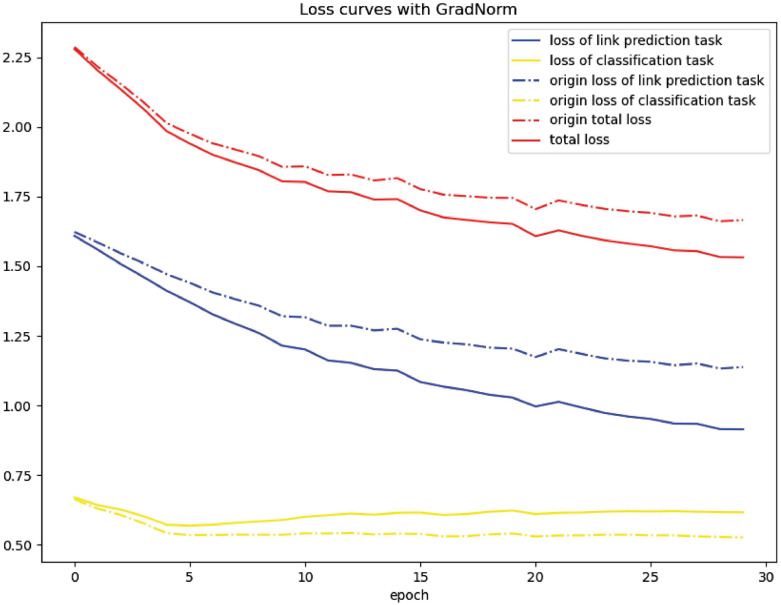
The adjusted loss curve and the origin loss curve of the two tasks.

To intuitively verify the effectiveness of GradNorm, we also plot the adjusted loss curves and the origin loss curves of the two tasks in Fig 7. As shown in the figure, the loss of the link prediction task is reduced by GradNorm, while the loss of the link multilabel classification task is increased, which is because the link prediction task uses more input data and has more parameters, and thus has a dominant influence on the whole model.

### 3.7 Case study

In this section, we aim to assess the proposed method’s ability to predict potential target genes for a specific type of TF in real-world scenarios. Specifically, we focus on the prediction lists for one particular TF to evaluate the recommendation performance. Specifically, we trained the KGE-TGI model on the entire set of known TF-target genes from the TRRUST database as the training dataset and restricted our analysis to the highest-ranked prediction for the specific TF of interest.

The aryl hydrocarbon receptor (AHR) is a transcription factor that plays a critical role in regulating the body’s response to environmental toxins and pollutants, such as dioxins, polycyclic aromatic hydrocarbons (PAHs), and other xenobiotic compounds. AhR is a cytosolic transcription factor that is normally inactive, bound to several co-chaperones. The top 10 target genes of the AHR are reported in Table 8. As shown in Table 8, 60% (6/10) of the predicted interactions were confirmed in the TRRUST dataset. We further searched relevant literature and found evidence that although the genes CRY2 and VEGFA were not recorded in TRRUST, they have been shown to have regulatory relationships with AHR in other studies, as indicated by the corresponding PMID numbers in the table. This further demonstrates the effectiveness of our proposed model.

**Table 8:** The top ten target genes of transcription factor AHR predicted by KGE-TGI model.

Gene	Score	Validation	PMID
VEGFA	5.7125	Unconfirmed	36347318
MYC	4.9240	Confirmed by TRRUST	/
CCND1	4.6253	Confirmed by TRRUST	/
RFC3	4.6149	Confirmed by TRRUST	/
MT2A	4.6149	Confirmed by TRRUST	/
GNAS	4.5727	Unconfirmed	/
CYP1A1	3.6712	Confirmed by TRRUST	/
C3	3.5834	Unconfirmed	/
CYP1B1	3.1916	Confirmed by TRRUST	/
CRY2	3.1217	Unconfirmed	277559298

## 4 Discussion

Predicting transcriptional regulation interaction is still a fundamental challenge, where the higher-order topological relationships of the entire gene regulatory network have not been well explored. In this work, we proposed KGE-TGI, a multi-task model using multi-subgraph convolution network for both predicting the existence and its type of transcriptional regulation interactions. A series of experiments were carried out on a real dataset constructed from three verified databases, including: TRRUST, DisGeNET and GENEONTOLOGY. We also used CTD database to provide chemical information as the node feature, and made a comprehensive analysis on the predicted results. The experimental results show that the KGE-TGI model has good performance and effectiveness on both tasks.

To the best of our knowledge, KGETGI is the first model capable of predicting both new potential transcriptional regulatory interactions and the regulatory types of those interactions simultaneously, and achieves the best performance on the TF-target gene interaction prediction task which consider the topology of the known transcription regulation network. Another main contribution is constructing a knowledge graph of transcription regulation, which comprehensively depicts the pattern of the known GRN. Moreover, KGE-TGI model is the first attempt to use a multi-subgraph convolution network architecture to extract and fuse the global information in the knowledge graph with the unique information on each subgraph. It has been proven that using knowledge graphs and multi-subgraph convolutional networks as improvements is effective, with a 9% increase in AUC on the transcriptional regulatory relationship prediction task compared to the GraphTGI model. Our future work will focus on how to construct transcriptional regulatory network knowledge graphs more effectively and accurately by integrating multi-omics information of genes, in order to infer transcriptional regulatory relationships with higher precision.
