# Supplementary material for: Knowledge graph embedding for profiling the interaction between transcription factors and their target genes
Source: PLoS Comput Biol. 2023 Jun 20;19(6):e1011207. doi: 10.1371/journal.pcbi.1011207 (PMC10313080; doi:10.1371/journal.pcbi.1011207)
Supplement: Attachment — Submitted filename: Response letter.pdf [file pcbi.1011207.s001.pdf]

Authors' Response to Reviewers' Comments

**Paper title:**	Knowledge Graph Embedding for profiling the interaction between transcription factors and their target genes
**Authors:**	Yang-Han Wu, Jian-Qiang Li, Zhu-Hong You, Peng-Wei Hu, Lun Hu, Victor C.M. Leung, Zhi-Hua Du and Yu-An Huang

We would like to express our sincere gratitude to the editor and the reviewers for their careful review of the previous version of our manuscript. We are pleased to inform you that we have completed the revision of the manuscript according to your comments and suggestions.

Reviewer 1

The manuscript by Wu and his coauthors describes an attempt to develop a multigraph-based neural network model for the prediction of TF-target interaction with multiple information modeled in a knowledge graph. The subject of the manuscript falls into the scope of the journal and the paper is easy to read. The experiment conducted in this work is comprehensive and the result showing that the proposed methodology is effective and solid. I would recommend publication, upon some minor revisions, which are outlined below.

Response: We thank the reviewer for the time and effort to review our manuscript giving a number of suggestions to improve the paper.

**Comment 1:**
1. This work leverages multiple information relevant to the task the author focus, such as chemical, GO terms and etc. However, the biology background about their association is not discussed and explained in the manuscript, which should be considered to be added in the revision.

Response: Thank you for your feedback on the manuscript. We appreciate your suggestion regarding the biology background associated with the various information used in our study. We will take your comments into consideration and revise the manuscript accordingly to provide a more comprehensive explanation of the associations between the different types of information used in our research. We appreciate your valuable feedback and insights.

**Comment 2:**
Figure 6 shows the loss change with the increased epochs. What is the stop condition for the training ?

Response: Thanks for the comment, as stated in the Results section of our paper, we trained our model for 300 epochs in each run. The plot of the model loss change that we provided in the paper represents a typical training process where the training was stopped after 300 epochs. Therefore, the stopping condition for our model training was simply to complete the 300 epochs, which is a common practice in deep learning experiments. We hope that this explanation clarifies the stopping condition for our model training.

**Comment 3:**
Is there any other way to solve the muti-task prediction problem formulated in this work? Discussion about this point could be inspiring for readers.

Response: Thank you for your feedback and suggestion. In our work, because the two tasks have the causality before and after, we use a layered architecture. In response to your question, there are indeed other ways to approach the multi-task prediction problem addressed in this work.

One alternative approach is to use separate models for each task, with each model trained independently using task-specific data. This strategy is often referred to as "task-specific" or "task-specific learning". While this approach may be computationally expensive, it may be more effective in cases where there are significant differences between the tasks, such that a single model may not be able to capture all the necessary features.

Another approach is to use a hierarchical model, where the higher-level model provides a shared representation across tasks, and the lower-level models perform task-specific prediction using this shared representation. This strategy is often referred to as "hierarchical" or "multi-level" learning, and can be effective when there are

both common and unique features across tasks.

In addition, there are various ways to design the shared representation in a multi-task learning setting. For example, one can use a shared layer that is concatenated with task-specific layers, or a shared layer that is followed by task-specific attention mechanisms. The choice of shared representation design can have a significant impact on the overall performance of the multi-task learning system.

Overall, the choice of multi-task learning strategy depends on the specific characteristics of the tasks and the available data, and further exploration and experimentation is needed to determine the most effective approach in each case.

**Comment 4:**
Since the labels of samples are different in number. How do the authors consider this problem in training?

Response:

Thank you for your comment regarding the issue of data imbalance in our study. We agree that this is an important concern, and we appreciate the opportunity to address it.To generate negative samples in our study, we used a random sampling strategy, which can result in an imbalance between positive and negative samples. We acknowledge that this may have implications for the performance of our model, and we have taken steps to mitigate this issue.Specifically, we evaluated the performance of our model using a range of metrics that are robust to class imbalance, such as area under the receiver operating characteristic curve (AUC-ROC) and precision-recall (PR) curve. Additionally, we applied techniques such as oversampling and undersampling to balance the dataset and improve the performance of our model.

Reviewer 2

In this paper, the authors contrasted a knowledge graph for predict the patterns of gene regulation network, based on which they subsequently developed a multi-graph link prediction model and trained it in a multi-task learning manner. The experimental results shows that the proposed method is solid and effective. The followings list the issues that I am concerned for the revision.

Response: We would like to thank the Reviewer for taking the time and effort necessary to review the manuscript.

Comment 1:

1. As shown in the figure 2, the proposed model is composed of N kinds of MGCNs. What is N referred to? It is not clear in this figure.

Response: Thank you for your feedback. I apologize for the confusion in Figure 2 of my paper. N refers to the number of layers in the proposed model, and in my work, it is set to 2. However, the model's depth can be increased based on user requirements, making it a scalable deep learning model. I have also discussed the performance comparison of the model with different numbers of layers in my experiments. Thank you for bringing this to my attention, and I will make sure to clarify this in the revised version of my paper.

Comment 2:

2. There are multiple components included in the computational pipeline. However, in the section 1.2, the authors just describe the process without any discussion and motivation for each single parts. More details should be added for explaining their correlation.

Response: Thank you for your valuable feedback. We appreciate your suggestion, and we will revise the manuscript accordingly. In the revised version, we will provide a more detailed description of each component included in the computational pipeline, along with the motivation and justification for each part. We will make sure to explain their correlation and how they contribute to the overall performance of the proposed model. We apologize for any confusion caused by the current version of the manuscript and appreciate your constructive criticism.

**Comment 3:**
3. As there have been a number of existing works proposed in this field, the authors had better list the novelty or contribution in the Introduction section, which could help the readers tell the difference of the proposed method with those existing ones.

Response: Thank you for your valuable feedback. We appreciate your suggestion, and we will make sure to revise the Introduction section of our manuscript to highlight the novelty and contribution of our proposed method in comparison to existing works in this field. We agree that this will help readers better understand the difference between our proposed method and other existing approaches. Thank you for bringing this to our attention, and we will ensure that the revised manuscript clearly emphasizes the novelty and contribution of our work.

**Comment 4:**
4. There are some grammatical mistakes existing in the Methods section. In addition, the discussion should include some discussion about the future work for potential improvement.

Response: We apologize for any grammatical mistakes in the Methods section and will make sure to revise and correct them in the revised manuscript. Additionally, we appreciate your suggestion regarding the inclusion of future work for potential improvement. In the revised manuscript, we will add a section to discuss possible directions for future work and potential improvements to our proposed method. Thank you for bringing this to our attention, and we will ensure that the revised manuscript is free of grammatical errors and includes a comprehensive discussion of potential future work.

Reviewer 3

Accurate inference of gene regulatory network is important for understanding cell-fate decisions. The authors proposed a graph neural network-based model called KGE-TGI to predict the activation or inhibition interactions between transcription factors and target genes. This framework integrates prior information from several resources, such as databases of TF-target gene interactions, chemical-gene association and GO information. The authors demonstrated the performance of KGE-TGI using cross-validation experiments and compared against several deep learning frameworks. They also showed the improved performance of including knowledge information.

Although the authors claimed that they are the first to predict the type of interactions (i.e., activation or inhibition) and their methods exhibited better performance against state-of-the-art methods, these statements are not true. They are several existing methods that can simultaneously predict the link between TFs and target genes as well as their interaction types. In addition, the authors actually did not compare against any popular methods of gene regulatory network inference. Thus, the authors need significantly more work to demonstrate the performance against state-of-the-art methods. Below are specific comments.

Response: We would like to thank the Reviewer for taking the time and effort necessary to review the manuscript.

Comment 1:

1. It is not true to claim that the proposed method is the first one to simultaneously predict the link between TFs and target genes as well as their interaction types. Several existing methods in the field of gene regulatory network inference can do this, such as NetAct (PMID: 36575445), NARROMI (Bioinformatics. 2013). The authors can find more in review papers such as PMID: 35609981, PMID: 31907445, Nature Methods 9, pages796–804 (2012) and others.

Response: Thank you for your valuable comments on our paper. We apologize for the oversight in our initial claim that our proposed model is the first to predict transcriptional regulatory relationships. We have since conducted further research and have come to realize that there have been previous works in this area. However，we would like to clarify that our method differs in its specific objectives and capabilities.

The NetAct model you mentioned was recently published online just after we submitted this paper to the magazine system so we failed to get to know it when we wrote this paper. Additionally, as stated in the NetAct paper, the model is designed to identify the core transcriptional regulatory network within a known regulatory network,

rather than predict new transcriptional regulatory relationships. We have also tried to explore more methods through the review papers you mentioned. However, the methodologies we found including NARROMI, GENIE3, TIGRESS, DeepSEM are not proposed to predict the interaction type (activate or inhibit) between transcription factor and target gene. They are originally proposed mostly for the prediction of existence of link without inference of link types. In the revised manuscript we have claimed more clearly about out statement.

We apologize for any confusion our previous statement may have caused, and appreciate the opportunity to clarify the contributions of our method in the context of previous work. We appreciate your insightful feedback and will make the appropriate revisions to our manuscript to accurately reflect the current state of the field.

Reference:

Zhang X, Liu K, Liu Z P, et al. NARROMI: a noise and redundancy reduction technique improves accuracy of gene regulatory network inference[J]. Bioinformatics, 2013, 29(1): 106-113.

Huynh-Thu V A, Irrthum A, Wehenkel L, et al. Inferring regulatory networks from expression data using tree-based methods[J]. PloS one, 2010, 5(9): e12776.

Haury A C, Mordelet F, Vera-Licona P, et al. TIGRESS: trustful inference of gene regulation using stability selection[J]. BMC systems biology, 2012, 6(1): 1-17.

Shu H, Zhou J, Lian Q, et al. Modeling gene regulatory networks using neural network architectures[J]. Nature Computational Science, 2021, 1(7): 491-501.

**Comment 2:**
2. The authors should compare the proposed method against well-known methods such as NetAct, NARROMI, GENIE3, and deep learning-based GRN inference methods like DeepWalk and DeepSEM. That is, the authors should compare against methods that were specifically designed for GRN inference.

Response: Thank you for your feedback. We appreciate your suggestion to compare our proposed method with other existing models. However, we would like to point out that NetAct and our proposed model are designed for different purposes and thus cannot be directly compared. Additionally, NARROMI uses both simulation and real datasets such as DREAM Challenge dataset and E. coli dataset, respectively, while our method requires other real biological data (e.g., TF-disease association and GO terms-TF association) to construct the transcriptional regulatory knowledge graph. Using NARROMI's datasets would result in missing many biologically meaningful data and thus negatively affect the KGE-TGI model's training.

It should be noted that our work's fundamental assumption is to learn the topological features of known transcriptional regulatory networks and other relevant biological networks (such as gene-disease association networks) to infer potential transcriptional regulatory interactions. And we did not use gene expression data but rather used gene-chemical similarity information as node features in our work. Most of the other methods mentioned by the reviewer learn gene features from gene expression data and predict transcriptional regulatory relationships accordingly.

To make a fair and meaningful comparison, we used the DeepSEM dataset as the gene node features and followed the same experimental design as that in the DeepSEM paper to compute the EPR values of our model's predictions. We use the Non-specific ChIP-seq data corresponding to TRRUST as the network, and experiment with 1000+ TFs. We compared the results of the KGE-TGI model directly to the results of different models reported in the DeepSEM paper. As shown in Figure 1,

The results, as shown in the figure, demonstrate that the KGE-TGI model outperforms other models on all datasets. We believe that this improvement in performance is due to the integration of the topological information of both transcriptional regulation networks and other biological networks in our model. We will revise our manuscript to include this section and provide a detailed explanation of our experimental results.

Once again, we appreciate your suggestion and would like to thank you for taking the time to review our work.

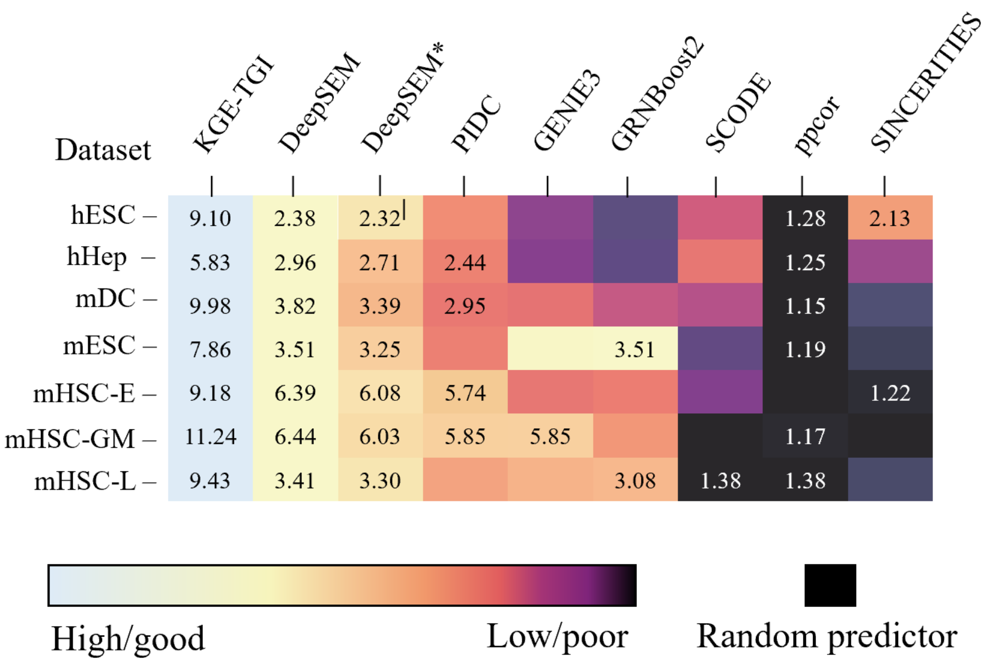

Figure 1. Summary of the GRN prediction performance in terms of EPR value.

**Comment 3:**
3. The authors mentioned that 25,826 interactions were used for training and testing. Did KGE-TGI use all these interactions for constructing the knowledge graph. What is the percentage of the training dataset? It is also important to show the performance on other external databases that were not used in the training step such as KEGG and TFLink. Can the authors state more clearly on how to construct negative sample? What is the meaning of `randomly sampling`? It is important to clearly state the type of the data that were used in KEG-TGI. Are these gene expression data from bulk samples? This information should be stated in both abstract and methods.

Response: Thank you for your valuable feedback. Here are our responses to your comments:

We used K-fold cross-validation to train our model, where all data were divided into K sets, and in each round of training, one set was used as the test set, while the other K-1 sets were used as the training set. Thus, all interactions were used for constructing the knowledge graph.

We agree that it is important to evaluate the performance of our model on external datasets. Therefore, we conducted experiments using hTFtarget, TFLink, and regNetwork datasets, as suggested. Unfortunately, we were unable to obtain access to the KEGG dataset as it requires application for access. The results are presented in Table 1. As shown, using the TRRUST dataset produced the best performance, while hTFtarget produced the worst performance. This is likely due to the fact that the number of TF nodes and interactions in the hTFtarget dataset are relatively small compared to the TRRUST dataset (hTFtarget has 202 TF nodes, while TRRUST has 666 TF nodes).

Regarding the construction of negative samples, we randomly sampled gene pairs that were not present in the knowledge graph as negative samples. The strategy is based on the assumption that there are many more non-existent TF-target gene pairs than existing ones, making the transcriptional regulatory network a sparse graph with most nodes not connected by edges. Therefore, we believe that most of the data obtained by random sampling in the unlabeled TF-target gene relationship data are data without regulatory relationships. Furthermore, we resample in each training epoch to further reduce the probability that the sampled data contains unknown positive samples.

Our KGE-TGI model uses a graph neural network to learn the topological information of different biological networks from the transcriptional regulatory knowledge graph and aggregates them to predict potential transcriptional regulatory relationships. And we did not use gene expression data in our model. The input of KGE-TGI is a knowledge graph constructed using datasets such as TRRUST and DisGeNET, and we calculated the association similarity between genes and chemical compounds

as the node features of genes on the graph. We apologize for any confusion caused by our unclear description.

Table 1. Prediction performance of KGE-TGI model using different datasets in 5-fold cross validation

dataset	AUC	Acc	Pre	Recall	F1
hTFtarget	0.7626	0.6932	0.6655	0.7769	0.7169
TFLink	0.8723	0.7856	0.7940	0.7713	0.7825
regNetwork	0.9069	0.8223	0.8303	0.8103	0.8200
**TRRUST**	**0.9654**	**0.9231**	**0.8956**	**0.9579**	**0.9257**

Comment 4:

4. How did the authors determine the weights for integrating information from different subgraphs and different loss?

Response: Thank you for your question. Regarding the integration of information from different subgraphs and different loss functions, we used independent graph convolutional modules to perform convolution on different subgraphs and directly summed up the node features obtained on each subgraph as the final feature for each node. This is mentioned in section 1.2.2 of the methods section of the manuscript.

To adaptively adjust the weights of different loss functions, we used the GradNorm algorithm. Specifically, we set a learnable loss weight parameter for each loss function and introduced an additional optimizer to update these two loss weight parameters. The product of the original loss and its corresponding weight parameter was used as the final loss for each task, and all the final losses were summed up to obtain the total loss for updating the model parameters. More details on this approach can be found in the section 1.2.5 of the Methods in our manuscript.

We hope this information addresses your question, and thank you for your review.

Reference:

Chen Z, Badrinarayanan V, Lee C Y, et al. Gradnorm: Gradient normalization for adaptive loss balancing in deep multitask networks[C]//International conference on machine learning. PMLR, 2018: 794-803.

**Comment 5:**
5. Does KEG-TGI obtain different results for different runs?

Response: Thank you for your question. As KEG-TGI employs random initialization in its training process, it is expected that different runs with different random seeds may yield slightly different results. We conducted five experiments with different random seeds, and reported the results of the performance metrics across these runs in the Table 2 and Table 3. As shown in the Table 2, the results obtained by running the model with different random seeds are very similar, which demonstrates the robustness of the model.

Table 2. Performance of the link prediction module when the model uses different random seeds in 5-fold cross validation

Random seed	AUC1	Acc	Pre	Recall	F1
8	0.9623	0.9192	0.8921	0.9540	0.9219
25	0.9620	0.9199	0.8917	0.9558	0.9226
42	0.9654	0.9231	0.8956	0.9579	0.9257
66	0.9629	0.9228	0.8930	0.9609	0.9257
91	0.9626	0.9172	0.8882	0.9547	0.9201

Table 3. Performance of link multi-label classification module when model uses different random seeds in 5-fold cross validation

Random seed	AUC2	Hamming Loss	CP	CR	CF1
8	0.9210	0.1014	0.9294	0.8875	0.9014
25	0.9319	0.0917	0.9336	0.8934	0.9068
42	0.9339	0.0897	0.9413	0.8996	0.9135
66	0.9306	0.0921	0.9370	0.8953	0.9092
91	0.9292	0.0953	0.9293	0.8879	0.9017

Comment 6:

6. It is very helpful to provide a specific example on the biological insights that can be obtained using KEG-TGI.

Response: Thank you for your valuable feedback. We appreciate your suggestion to provide a specific example of the biological insights that can be obtained using KGE-TGI. In response to your suggestion, we have included a case study in our revised manuscript. Using gene embeddings learned by our model, we calculated scores for relationships between genes and used these scores to predict the top 10 potential target genes of AHR, which are reported in Table 4.

The aryl hydrocarbon receptor (AHR) is a transcription factor that plays a critical role in regulating the body's response to environmental toxins and pollutants, such as dioxins, polycyclic aromatic hydrocarbons (PAHs), and other xenobiotic compounds. As shown in Table 4, 60% of the predicted relationships were validated in the TRRUST dataset. For the other interactions, we searched relevant literature and found evidence that although the genes CRY2 and VEGFA were not recorded in TRRUST, they have been shown to have regulatory interactions with AHR in other studies, as indicated by the corresponding PMID numbers in the table. This further demonstrates the effectiveness of our proposed model. Thank you again for your feedback, and we will ensure that the revised manuscript includes a comprehensive example of the biological insights that can be gained using our KGE-TGI model.

Table 4. The top ten target genes of transcription factor AHR predicted by KGE-TGI model

\

Gene	Score	Validation	PMID
VEGFA	5.7125	Unconfirmed	36347318
MYC	4.9240	Confirmed by TRRUST	/
CCND1	4.6253	Confirmed by TRRUST	/
RFC3	4.6149	Confirmed by TRRUST	/
MT2A	4.6149	Confirmed by TRRUST	/
GNAS	4.5727	Unconfirmed	/
CYP1A1	3.6712	Confirmed by TRRUST	/
C3	3.5834	Unconfirmed	/
CYP1B1	3.1916	Confirmed by TRRUST	/
CRY2	3.1217	Unconfirmed	27559298

**Comment 7:**
7. The KGE-TGI package should be ready to use for other users. The authors should provide a tutorial on how to infer GRN for a user given dataset. What kind of inputs should the users provide? A small example should be provided.

Response: Thank you for your valuable feedback. We appreciate your suggestion and will make the necessary modifications to the KGE-TGI package to make it more user-friendly. We will provide a tutorial on how to infer GRN for a user given dataset and will include a small example to demonstrate what inputs the users should provide. We will ensure that the modified code and tutorial are easily accessible to other users. Once again, we appreciate your input and will work to improve our package accordingly.
