# Supplementary material for: Knowledge graph embedding for profiling the interaction between transcription factors and their target genes
Source: PLoS Comput Biol. 2023 Jun 20;19(6):e1011207. doi: 10.1371/journal.pcbi.1011207 (PMC10313080; doi:10.1371/journal.pcbi.1011207)
Supplement: Attachment — Submitted filename: Response letter.docx [file pcbi.1011207.s002.docx]

**Authors’ Response to Reviewers’ Comments**

| **Paper title:** | Knowledge Graph Embedding for profiling the interaction between transcription factors and their target genes |
| --- | --- |
| **Authors:** | Yang-Han Wu, Yu-An Huang, Jian-Qiang Li, Zhu-Hong You, Peng-Wei Hu, Lun Hu, Victor C.M. Leung, and Zhi-Hua Du |

We would like to express our sincere gratitude to the editor and the reviewers for their careful review of the previous version of our manuscript. We are pleased to inform you that we have completed the revision of the manuscript according to your comments and suggestions.

Reviewer 1

*No.*

***Response:*** We thank the reviewer for the time and effort to review our manuscript, we are glad to hear that our previous responses have addressed your concerns.

Reviewer 2

*The authors have answered all my questions.*

***Response:*** We are glad to hear that our previous responses have addressed all queries of reviewer.

Reviewer 3

*The authors have addressed most of my concerns. There are a few of them to be further addressed.*

***Response:*** We would like to thank the Reviewer for taking the time and effort necessary to review the manuscript.

Comment 1:

*1.* *The authors have addressed some of my concerns, but they did not make corrections in the manuscript.*

*(1) The authors should also modify the statement in the Abstract: “There is still no computational method available to predict them.”*

*(2)* *It is important to clarify in the Abstract that KGE-TGI is not based on any expression data, but dependent on the topology information from several resources. Therefore, KGE-TGI is not able to infer expression data-driven links.*

*(3)* *The authors should make changes on their package based on my suggestions before further consideration of the manuscript.*

***Response:*** Thank you for your valuable feedback on our manuscript. We appreciate your comments and suggestions, and we have addressed some of your concerns.

**Q**: The authors should also modify the statement in the Abstract: “There is still no computational method available to predict them.”

**A**: We apologize for not making the necessary corrections in the manuscript earlier. We have now modified the statement in the Abstract as per your suggestion, and it reads: "Although several computational methods exist to predict gene interactions and their type, there is still no method available to predict them solely based on topology information."

**Q**: It is important to clarify in the Abstract that KGE-TGI is not based on any expression data, but dependent on the topology information from several resources. Therefore, KGE-TGI is not able to infer expression data-driven links.

**A**: We agree with your suggestion and have updated the Abstract to clarify that KGE-TGI is not based on gene expression data, but relies on topology information. We hope this clarification addresses your concern.

**Q**: The authors should make changes on their package based on my suggestions before further consideration of the manuscript.

**A**: We appreciate your valuable feedback and have carefully considered your suggestions for changes to our package. We will make the necessary modifications based on your recommendations before resubmitting the manuscript. We apologize for any inconvenience caused and thank you again for your time and effort in reviewing our work.

Once again, we appreciate your comments, and we hope that the revised manuscript meets your expectations.

Comment 2:

*2.* *When comparing different methods, why do not use the metrics in Fig. 4? These metrics were widely used in the original manuscript, which is much better than the EPR value. The authors should also describe how different methods were implemented for comparisons?* *What is the input of different methods? What is the DeepSEm*? Because the authors have compared their method against methods such as PIDC and GENIE3, it should be able to also evaluate the performance of NetAct and NARROMI based on the gene expression data.*

***Response:*** Thank you for your valuable comments.

**Q**: *When comparing different methods, why do not use the metrics in Fig. 4?* *These metrics were widely used in the original manuscript, which is much better than the EPR value.*

**A**: We apologize for the confusion and oversight on our part. In our previous experiments, we followed the comparison method used in the DeepSEM paper, which led us to use the EPR value as the evaluation metric instead of the metrics in Fig. 4. However, we agree with your suggestion that the metrics in Fig. 4 are more appropriate for comparing different methods. Due to space limitations, we only used the AUC metric from the metrics used in Fig. 4 to compare the performance of different models. In addition, calculating other metrics for these models, such as accuracy and precision, requires setting a threshold, and performance is sensitive to different thresholds. In all experiments in the original manuscript, we chose a threshold of 0.5 when calculating the relevant metrics for the KGE-TGI model. However, the optimal threshold for these models was not specified in the related papers, so we did not use accuracy, precision, and other metrics for comparison. AUC value takes into account the trade-off between true positive rate (sensitivity) and false positive rate (1-specificity) across different probability thresholds, providing a more comprehensive evaluation of model performance that is less affected by class imbalance. The AUC comparison results of KGE-TGI and other models are shown in Figure 1. As shown in Figure 1, the KGETGI model outperformed the other models. AUC values marked in dark color, which are less than 0.5, indicate that the model performs worse than random guessing on the dataset. We appreciate your insightful comments, and we will revise our manuscript accordingly.

Figure 1. Summary of the GRN prediction performance in terms of AUC metric.

**Q**: The authors should also describe how different methods were implemented for comparisons?

**A**: Thank you for your feedback. We appreciate your suggestion to describe how different methods were implemented for comparison. To compare our KGE-TGI model with other methods, we directly used the experimental results reported in the GENELink model paper, and the implementation was performed according to the description in the GENELink paper. We apologize for any confusion caused by not providing this information in the manuscript and will revise the paper to include this description. Thank you again for your valuable feedback.

**Q**: What is the input of different methods?

**A**: In this comparative experiment, the input for GNE, DeepSEM, PCC, MI, SCODE, GRNBoost2 and GENIE3 models is gene expression data matrix, where each row represents a gene and each column represents a cell. For GENELink, both the gene expression data matrix and prior adjacency matrix information need to be input simultaneously. In this comparison experiment, the KGE-TGI model used a complex graph data constructed from the TRRUST dataset and other datasets as the input for the graph structure and used gene expression data as node features. For nodes with missing gene expression data, we calculated the average of all gene expression data as their feature. Specifically, the gene expression data used by all models were cell-type non-specific ChIP-seq data, including: human embryonic stem cells (hESC), human mature hepatocytes (hHep), mouse dendritic cells (mDC), mouse embryonic stem (mESC), mouse erythroid lineage (mHSC-E), mouse granulocyte-macrophage lineage (mHSC-GM) and mouse lymphoid lineage (mHSC-L).

**Q**: What is the DeepSEm*?

**A**: DeepSEM* denotes the DeepSEM model without using ensemble learning. We apologize for the lack of clarity in our description about DeepSEM* and have removed its comparison results, only comparing the DeepSEM model.

**Q**: Because the authors have compared their method against methods such as PIDC and GENIE3, it should be able to also evaluate the performance of NetAct and NARROMI based on the gene expression data.

**A**: We regret that we were unable to include NARROMI and NetAct in our comparative experiments. The purpose of NetAct is to identify the core gene regulatory circuit that governs the decision-making of a biological process, rather than predicting new transcriptional regulatory relationships, which is the primary objective of the KGE-TGI model. Therefore, we decided not to include NetAct in our comparison. Additionally, the webpage (http://csb.shu.edu.cn/narromi.html) that provides the implementation code of the NARROMI model in the NARROMI paper is currently inaccessible, which prevented us from including it in our comparison. Nonetheless, we appreciate your feedback and will take it into account for future studies.

We have made the corresponding revisions to the manuscript regarding the feedback, and we thank you again for your helpful comments.
