# Supplementary material for: Knowledge graph embedding for profiling the interaction between transcription factors and their target genes
Source: PLoS Comput Biol. 2023 Jun 20;19(6):e1011207. doi: 10.1371/journal.pcbi.1011207 (PMC10313080; doi:10.1371/journal.pcbi.1011207)
Supplement: Peer review history

# Peer review: Decision Letter 0

**DOI:** 10.1371/journal.pcbi.1011207.r001

**Authors:** Qing Nie, Kiran Raosaheb Patil

23 Jan 2023

Dear dr. Huang,

Thank you very much for submitting your manuscript "Knowledge Graph Embedding for profiling the interaction between transcription factors and their target genes" for consideration at PLOS Computational Biology.

As with all papers reviewed by the journal, your manuscript was reviewed by members of the editorial board and by several independent reviewers. In light of the reviews (below this email), we would like to invite the resubmission of a significantly-revised version that takes into account the reviewers' comments.

Please also ensure code availability as per PLOS requirements.

We cannot make any decision about publication until we have seen the revised manuscript and your response to the reviewers' comments. Your revised manuscript is also likely to be sent to reviewers for further evaluation.

When you are ready to resubmit, please upload the following:

[1] A letter containing a detailed list of your responses to the review comments and a description of the changes you have made in the manuscript. Please note while forming your response, if your article is accepted, you may have the opportunity to make the peer review history publicly available. The record will include editor decision letters (with reviews) and your responses to reviewer comments. If eligible, we will contact you to opt in or out.

[2] Two versions of the revised manuscript: one with either highlights or tracked changes denoting where the text has been changed; the other a clean version (uploaded as the manuscript file).

Important additional instructions are given below your reviewer comments.

Please prepare and submit your revised manuscript within 60 days. If you anticipate any delay, please let us know the expected resubmission date by replying to this email. Please note that revised manuscripts received after the 60-day due date may require evaluation and peer review similar to newly submitted manuscripts.

Thank you again for your submission. We hope that our editorial process has been constructive so far, and we welcome your feedback at any time. Please don't hesitate to contact us if you have any questions or comments.

Sincerely,

Qing Nie

Academic Editor

PLOS Computational Biology

Kiran Patil

Section Editor

PLOS Computational Biology

***********************

Reviewer's Responses to Questions

**Comments to the Authors:**

**Please note here if the review is uploaded as an attachment.**

Reviewer #1: The manuscript by Wu and his coauthors describes an attempt to develop a multigraph-based neural network model for the prediction of TF-target interaction with multiple information modeled in a knowledge graph. The subject of the manuscript falls into the scope of the journal and the paper is easy to read. The experiment conducted in this work is comprehensive and the result showing that the proposed methodology is effective and solid. I would recommend publication, upon some minor revisions, which are outlined below.

1. This work leverages multiple information relevant to the task the author focus, such as chemical, GO terms and etc. However, the biology background about their association is not discussed and explained in the manuscript, which should be considered to be added in the revision.

2. Figure 6 shows the loss change with the increased epochs. What is the stop condition for the training？

3. Is there any other way to solve the muti-task prediction problem formulated in this work? Discussion about this point could be inspiring for readers.

4. Since the labels of samples are different in number. How do the authors consider this problem in training?

Reviewer #2: In this paper, the authors contrasted a knowledge graph for predict the patterns of gene regulation network, based on which they subsequently developed a multi-graph link prediction model and trained it in a multi-task learning manner. The experimental results shows that the proposed method is solid and effective. The followings list the issues that I am concerned for the revision.

1. As shown in the figure 2, the proposed model is composed of N kinds of MGCNs. What is N referred to? It is not clear in this figure.

2. There are multiple components included in the computational pipeline. However, in the section 1.2, the authors just describe the process without any discussion and motivation for each single parts. More details should be added for explaining their correlation.

3. As there have been a number of existing works proposed in this field, the authors had better list the novelty or contribution in the Introduction section, which could help the readers tell the difference of the proposed method with those existing ones.

4. There are some grammatical mistakes existing in the Methods section. In addition, the discussion should include some discussion about the future work for potential improvement.

Reviewer #3: Accurate inference of gene regulatory network is important for understanding cell-fate decisions. The authors proposed a graph neural network-based model called KGE-TGI to predict the activation or inhibition interactions between transcription factors and target genes. This framework integrates prior information from several resources, such as databases of TF-target gene interactions, chemical-gene association and GO information. The authors demonstrated the performance of KGE-TGI using cross-validation experiments and compared against several deep learning frameworks. They also showed the improved performance of including knowledge information.

Although the authors claimed that they are the first to predict the type of interactions (i.e., activation or inhibition) and their methods exhibited better performance against state-of-the-art methods, these statements are not true. They are several existing methods that can simultaneously predict the link between TFs and target genes as well as their interaction types. In addition, the authors actually did not compare against any popular methods of gene regulatory network inference. Thus, the authors need significantly more work to demonstrate the performance against state-of-the-art methods. Below are specific comments.

1. It is not true to claim that the proposed method is the first one to simultaneously predict the link between TFs and target genes as well as their interaction types. Several existing methods in the field of gene regulatory network inference can do this, such as NetAct (PMID: 36575445), NARROMI (Bioinformatics. 2013). The authors can find more in review papers such as PMID: 35609981, PMID: 31907445, Nature Methods 9, pages796–804 (2012) and others.

2. The authors should compare the proposed method against well-known methods such as NetAct, NARROMI, GENIE3, and deep learning-based GRN inference methods like DeepWalk and DeepSEM. That is, the authors should compare against methods that were specifically designed for GRN inference.

3. The authors mentioned that 25,826 interactions were used for training and testing. Did KGE-TGI use all these interactions for constructing the knowledge graph. What is the percentage of the training dataset? It is also important to show the performance on other external databases that were not used in the training step such as KEGG and TFLink. Can the authors state more clearly on how to construct negative sample? What is the meaning of `randomly sampling`? It is important to clearly state the type of the data that were used in KEG-TGI. Are these gene expression data from bulk samples? This information should be stated in both abstract and methods.

4. How did the authors determine the weights for integrating information from different subgraphs and different loss?

5. Does KEG-TGI obtain different results for different runs?

6. It is very helpful to provide a specific example on the biological insights that can be obtained using KEG-TGI.

7. The KGE-TGI package should be ready to use for other users. The authors should provide a tutorial on how to infer GRN for a user given dataset. What kind of inputs should the users provide? A small example should be provided.

**********

**Have the authors made all data and (if applicable) computational code underlying the findings in their manuscript fully available?**

The PLOS Data policy requires authors to make all data and code underlying the findings described in their manuscript fully available without restriction, with rare exception (please refer to the Data Availability Statement in the manuscript PDF file). The data and code should be provided as part of the manuscript or its supporting information, or deposited to a public repository. For example, in addition to summary statistics, the data points behind means, medians and variance measures should be available. If there are restrictions on publicly sharing data or code —e.g. participant privacy or use of data from a third party—those must be specified.

Reviewer #1: None

Reviewer #2: Yes

Reviewer #3: **No: **The computational codes underlying the findings are not fully available.

**********

PLOS authors have the option to publish the peer review history of their article (what does this mean?). If published, this will include your full peer review and any attached files.

If you choose “no”, your identity will remain anonymous but your review may still be made public.

**Do you want your identity to be public for this peer review?** For information about this choice, including consent withdrawal, please see our Privacy Policy.

Reviewer #1: No

Reviewer #2: No

Reviewer #3: No

Figure Files:

While revising your submission, please upload your figure files to the Preflight Analysis and Conversion Engine (PACE) digital diagnostic tool, https://pacev2.apexcovantage.com. PACE helps ensure that figures meet PLOS requirements. To use PACE, you must first register as a user. Then, login and navigate to the UPLOAD tab, where you will find detailed instructions on how to use the tool. If you encounter any issues or have any questions when using PACE, please email us at figures@plos.org.

Data Requirements:

Please note that, as a condition of publication, PLOS' data policy requires that you make available all data used to draw the conclusions outlined in your manuscript. Data must be deposited in an appropriate repository, included within the body of the manuscript, or uploaded as supporting information. This includes all numerical values that were used to generate graphs, histograms etc.. For an example in PLOS Biology see here: http://www.plosbiology.org/article/info%3Adoi%2F10.1371%2Fjournal.pbio.1001908#s5.

Reproducibility:

To enhance the reproducibility of your results, we recommend that you deposit your laboratory protocols in protocols.io, where a protocol can be assigned its own identifier (DOI) such that it can be cited independently in the future. Additionally, PLOS ONE offers an option to publish peer-reviewed clinical study protocols. Read more information on sharing protocols at https://plos.org/protocols?utm_medium=editorial-email&utm_source=authorletters&utm_campaign=protocols

# Author comment: Author response to Decision Letter 0

**DOI:** 10.1371/journal.pcbi.1011207.r002

31 Mar 2023

Attachment Submitted filename: Response letter.pdf Click here for additional data file.

# Peer review: Decision Letter 1

**DOI:** 10.1371/journal.pcbi.1011207.r003

**Authors:** Qing Nie, Kiran Raosaheb Patil

17 Apr 2023

Dear dr. Huang,

Thank you very much for submitting your manuscript "Knowledge Graph Embedding for profiling the interaction between transcription factors and their target genes" for consideration at PLOS Computational Biology. As with all papers reviewed by the journal, your manuscript was reviewed by members of the editorial board and by several independent reviewers. The reviewers appreciated the attention to an important topic. Based on the reviews, we are likely to accept this manuscript for publication, providing that you modify the manuscript according to the review recommendations.

Please prepare and submit your revised manuscript within 30 days. If you anticipate any delay, please let us know the expected resubmission date by replying to this email.

When you are ready to resubmit, please upload the following:

[1] A letter containing a detailed list of your responses to all review comments, and a description of the changes you have made in the manuscript. Please note while forming your response, if your article is accepted, you may have the opportunity to make the peer review history publicly available. The record will include editor decision letters (with reviews) and your responses to reviewer comments. If eligible, we will contact you to opt in or out

[2] Two versions of the revised manuscript: one with either highlights or tracked changes denoting where the text has been changed; the other a clean version (uploaded as the manuscript file).

Important additional instructions are given below your reviewer comments.

Thank you again for your submission to our journal. We hope that our editorial process has been constructive so far, and we welcome your feedback at any time. Please don't hesitate to contact us if you have any questions or comments.

Sincerely,

Qing Nie

Academic Editor

PLOS Computational Biology

Kiran Patil

Section Editor

PLOS Computational Biology

***********************

A link appears below if there are any accompanying review attachments. If you believe any reviews to be missing, please contact ploscompbiol@plos.org immediately:

Reviewer's Responses to Questions

**Comments to the Authors:**

**Please note here if the review is uploaded as an attachment.**

Reviewer #1: no

Reviewer #2: The authors have answered all my questions.

Reviewer #3: The authors have addressed most of my concerns. There are a few of them to be further addressed.

1. The authors have addressed some of my concerns, but they did not make corrections in the manuscript.

(1) The authors should also modify the statement in the Abstract: “There is still no computational method available to predict them.”

(2) It is important to clarify in the Abstract that KGE-TGI is not based on any expression data, but dependent on the topology information from several resources. Therefore, KGE-TGI is not able to infer expression data-driven links.

(3) The authors should make changes on their package based on my suggestions before further consideration of the manuscript.

2. When comparing different methods, why do not use the metrics in Fig. 4? These metrics were widely used in the original manuscript, which is much better than the EPR value. The authors should also describe how different methods were implemented for comparisons? What is the input of different methods? What is the DeepSEm*? Because the authors have compared their method against methods such as PIDC and GENIE3, it should be able to also evaluate the performance of NetAct and NARROMI based on the gene expression data.

**********

**Have the authors made all data and (if applicable) computational code underlying the findings in their manuscript fully available?**

The PLOS Data policy requires authors to make all data and code underlying the findings described in their manuscript fully available without restriction, with rare exception (please refer to the Data Availability Statement in the manuscript PDF file). The data and code should be provided as part of the manuscript or its supporting information, or deposited to a public repository. For example, in addition to summary statistics, the data points behind means, medians and variance measures should be available. If there are restrictions on publicly sharing data or code —e.g. participant privacy or use of data from a third party—those must be specified.

Reviewer #1: None

Reviewer #2: Yes

Reviewer #3: **No: **

**********

PLOS authors have the option to publish the peer review history of their article (what does this mean?). If published, this will include your full peer review and any attached files.

If you choose “no”, your identity will remain anonymous but your review may still be made public.

**Do you want your identity to be public for this peer review?** For information about this choice, including consent withdrawal, please see our Privacy Policy.

Reviewer #1: **Yes: **Quan Zou

Reviewer #2: No

Reviewer #3: No

Figure Files:

While revising your submission, please upload your figure files to the Preflight Analysis and Conversion Engine (PACE) digital diagnostic tool, https://pacev2.apexcovantage.com. PACE helps ensure that figures meet PLOS requirements. To use PACE, you must first register as a user. Then, login and navigate to the UPLOAD tab, where you will find detailed instructions on how to use the tool. If you encounter any issues or have any questions when using PACE, please email us at figures@plos.org.

Data Requirements:

Please note that, as a condition of publication, PLOS' data policy requires that you make available all data used to draw the conclusions outlined in your manuscript. Data must be deposited in an appropriate repository, included within the body of the manuscript, or uploaded as supporting information. This includes all numerical values that were used to generate graphs, histograms etc.. For an example in PLOS Biology see here: http://www.plosbiology.org/article/info%3Adoi%2F10.1371%2Fjournal.pbio.1001908#s5.

Reproducibility:

To enhance the reproducibility of your results, we recommend that you deposit your laboratory protocols in protocols.io, where a protocol can be assigned its own identifier (DOI) such that it can be cited independently in the future. Additionally, PLOS ONE offers an option to publish peer-reviewed clinical study protocols. Read more information on sharing protocols at https://plos.org/protocols?utm_medium=editorial-email&utm_source=authorletters&utm_campaign=protocols

References:

Review your reference list to ensure that it is complete and correct. If you have cited papers that have been retracted, please include the rationale for doing so in the manuscript text, or remove these references and replace them with relevant current references. Any changes to the reference list should be mentioned in the rebuttal letter that accompanies your revised manuscript.

*If you need to cite a retracted article, indicate the article’s retracted status in the References list and also include a citation and full reference for the retraction notice.*

# Author comment: Author response to Decision Letter 1

**DOI:** 10.1371/journal.pcbi.1011207.r004

19 May 2023

Attachment Submitted filename: Response letter.docx Click here for additional data file.

# Editor report: Decision Letter 2

**DOI:** 10.1371/journal.pcbi.1011207.r005

**Authors:** Qing Nie, Kiran Raosaheb Patil

23 May 2023

Dear dr. Huang,

We are pleased to inform you that your manuscript 'Knowledge Graph Embedding for profiling the interaction between transcription factors and their target genes' has been provisionally accepted for publication in PLOS Computational Biology.

Before your manuscript can be formally accepted you will need to complete some formatting changes, which you will receive in a follow up email. A member of our team will be in touch with a set of requests.

Please note that your manuscript will not be scheduled for publication until you have made the required changes, so a swift response is appreciated.

IMPORTANT: The editorial review process is now complete. PLOS will only permit corrections to spelling, formatting or significant scientific errors from this point onwards. Requests for major changes, or any which affect the scientific understanding of your work, will cause delays to the publication date of your manuscript.

Should you, your institution's press office or the journal office choose to press release your paper, you will automatically be opted out of early publication. We ask that you notify us now if you or your institution is planning to press release the article. All press must be co-ordinated with PLOS.

Thank you again for supporting Open Access publishing; we are looking forward to publishing your work in PLOS Computational Biology. 

Best regards,

Qing Nie

Academic Editor

PLOS Computational Biology

Kiran Patil

Section Editor

PLOS Computational Biology

***********************************************************

# Editor report: Acceptance letter

**DOI:** 10.1371/journal.pcbi.1011207.r006

**Authors:** Qing Nie, Kiran Raosaheb Patil

16 Jun 2023

PCOMPBIOL-D-22-01903R2 

Knowledge Graph Embedding for profiling the interaction between transcription factors and their target genes

Dear Dr Huang,

I am pleased to inform you that your manuscript has been formally accepted for publication in PLOS Computational Biology. Your manuscript is now with our production department and you will be notified of the publication date in due course.

The corresponding author will soon be receiving a typeset proof for review, to ensure errors have not been introduced during production. Please review the PDF proof of your manuscript carefully, as this is the last chance to correct any errors. Please note that major changes, or those which affect the scientific understanding of the work, will likely cause delays to the publication date of your manuscript. 

Soon after your final files are uploaded, unless you have opted out, the early version of your manuscript will be published online. The date of the early version will be your article's publication date. The final article will be published to the same URL, and all versions of the paper will be accessible to readers.

Thank you again for supporting PLOS Computational Biology and open-access publishing. We are looking forward to publishing your work! 

With kind regards,

Zsofi Zombor

PLOS Computational Biology | Carlyle House, Carlyle Road, Cambridge CB4 3DN | United Kingdom ploscompbiol@plos.org | Phone +44 (0) 1223-442824 | ploscompbiol.org | @PLOSCompBiol
